# Spontaneous time-reversal symmetry breaking in twisted double bilayer graphene

**DOI:** 10.1038/s41467-022-34192-x

**Published:** 2022-10-29

**Authors:** Manabendra Kuiri, Christopher Coleman, Zhenxiang Gao, Aswin Vishnuradhan, Kenji Watanabe, Takashi Taniguchi, Jihang Zhu, Allan H. MacDonald, Joshua Folk

**Affiliations:** 1 Department of Physics and Astronomy & Stewart Blusson Quantum Matter Institute, University of British Columbia, Vancouver, BC V6T 1Z4 Canada; 2 Research Center for Functional Materials, National Institute for Materials Science, Namiki 1-1, Tsukuba, Ibaraki 305-0044 Japan; 3 International Center for Materials Nanoarchitectonics, National Institute for Materials Science, Namiki 1-1, Tsukuba, Ibaraki, Japan; 4 Physics Department, University of Texas at Austin, Austin, TX 78712 USA

**Keywords:** Electronic properties and materials, Surfaces, interfaces and thin films

## Abstract

Twisted double bilayer graphene (tDBG) comprises two Bernal-stacked bilayer graphene sheets with a twist between them. Gate voltages applied to top and back gates of a tDBG device tune both the flatness and topology of the electronic bands, enabling an unusual level of experimental control. Metallic states with broken spin and valley symmetries have been observed in tDBG devices with twist angles in the range 1.2–1.3°, but the topologies and order parameters of these states have remained unclear. We report the observation of an anomalous Hall effect in the correlated metal state of tDBG, with hysteresis loops spanning hundreds of mT in out-of-plane magnetic field (*B*_⊥_) that demonstrate spontaneously broken time-reversal symmetry. The *B*_⊥_ hysteresis persists for in-plane fields up to several Tesla, suggesting valley (orbital) ferromagnetism. At the same time, the resistivity is strongly affected by even mT-scale values of in-plane magnetic field, pointing to spin-valley coupling or to a direct orbital coupling between in-plane field and the valley degree of freedom.

## Abstract (web summary)

Twisted double bilayer graphene (tDBG) comprises two Bernal-stacked bilayer graphene sheets with a twist between them. Here, the authors report a strong anomalous Hall effect in the correlated-metal regime of tDBG, indicating time reversal symmetry breaking from orbital ferromagnetism, likely associated with valley polarization.

## Introduction

The interplay between band topology and Coulomb interactions has emerged as a frontier in the study of two-dimensional (2D) materials with flat electronic bands^1–5^. Graphene-based van der Waals (vdW) heterostructures offer a flexible platform from which to investigate this interplay, due to their native Dirac points close to the Fermi level that provide a resource for band topology. Flat bands may be engineered by clever heterostructure design^1,2,6^ and tuned using experimental knobs such as magnetic field or gate voltage^7,8^; the topology of the resulting bands is then readily controlled, whether by choice of heterostructure stacking or by tuning applied gate voltages^9,10^. Starting from flat bands, Coulomb interactions often lead to spontaneous symmetry breaking, yielding exotic electronic phases such as fractional Chern insulators^11^ or unconventional superconductivity^2,12^.

The physical phenomenology that results from broken symmetry phases depends on the topology of the underlying electronic bands^5,13^. For example, the anomalous Hall effect (AHE) is a striking experimental signature that emerges due to spontaneously broken time reversal symmetry associated with spin or valley polarization. This broken time reversal symmetry creates an “anomalous” Hall resistivity when transport is via bands with finite Berry curvature^14^. AHE reflecting orbital ferromagnetism has been reported in multi-layer vdW heterostructures such as twisted bilayer graphene aligned with hexagonal boron nitride (*h*BN)^15,16^, and in naturally-occurring structures like Bernal-stacked (AB) bilayer graphene^17^.

**Fig. 1:**
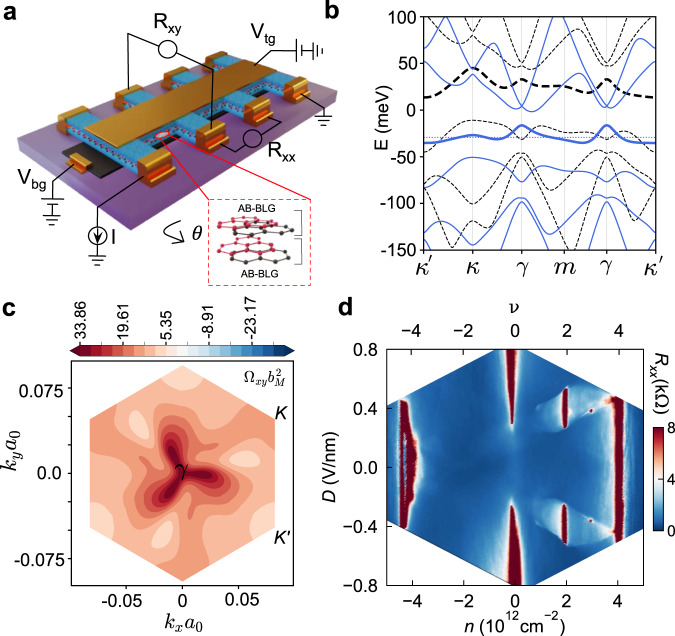
Twisted double bilayer graphene. **a** Schematic of a tDBG device consisting of two Bernal (AB) bilayer graphene layers stacked with a twist angle *θ* ≈ 1. 3°. The stack is encapsulated with top and bottom hexagonal boron nitride (*h*BN) layers, with a graphite bottom-gate (*V*_*bg*_) and a metal top-gate (*V*_*tg*_) to independently tune the density (*n*) and displacement field (*D*). **b** Calculated moiré band dispersion for tDBG with twist angle *θ* = 1. 3° and *D* = 0.44 V/nm. The solid (dashed) line corresponds to Hartree-Fock (single particle) calculations, with thicker lines indicating the first conduction band. HF calculations are filling-dependent, and are made for *ν* = 3.7. **c** Berry curvature (**Ω**) calculated for the conduction band. **d** Four terminal resistance, *R*_*xx*_, as a function of *n* and *D* at *T* = 20mK, ***B*** = 0 for D1. The top axis shows the band filling, *ν*.

Twisted double bilayer graphene (tDBG)—two AB-stacked bilayer graphene sheets misaligned by twist angle *θ*—is a uniquely tunable system in which band topology, correlations and broken symmetry phases can be manipulated using top- and back-gate voltages to control the electrostatic doping, *n*, and vertical displacement field, *D* (Fig. 1a)^9,10,18^. When *θ* ~ 1. 3°, the moiré-modified conduction band (hereafter referred to as the moiré band) can be tuned using *D* to be nearly flat, separated from a dispersive band at higher energy and from the valence band below (Fig. 1b)^19^. For gate voltages that place the Fermi energy within such a flat and isolated moiré band, transport measurements typically report regions of higher resistivity sometimes referred to as ‘haloes’. Theoretical predictions^9,10,20^ and experimental data^19,21–28^ indicate that, within the halo regions, the moiré band is topologically non-trivial and interactions lead to broken spin and valley symmetries, similar to the symmetry breaking observed in quantum Hall ferromagnetism^29,30^.

The precise way in which spin and valley symmetries are broken in tDBG remains an open problem^10,19,21,24^. Valley-polarized, spin-valley-locked, spin-polarized, and intervalley coherent states have all been considered^10^. The consistent observation of an insulating state when the moiré band is half filled provides a valuable clue. The resistance of this state increases with in-plane field, *B*_∥_, suggesting that the metallic states on either side may be spin polarized^21,22,24^ but leaving the question of possible valley order unaddressed^19,24,31^. In particular, an AHE of the kind reported in other graphene systems has not been reported in tDBG with small *θ*.

Here, we report the observation of a strong AHE in AB-AB stacked tDBG (*θ* = 1. 3°), with longitudinal (*R*_xx_) and Hall resistance (*R*_*xy*_) that are sharply hysteretic under out-of-plane magnetic field (*B*_⊥_). The data demonstrate orbital (valley) time reversal symmetry breaking, which modifies the Hall resistivity when there is strong Berry curvature^14^, confirming predictions for tDBG near the top of the moiré band (Fig. 1c). Qualitative differences between in- and out-of-plane magnetic field dependence in the AHE signatures reinforce the orbital (valley) character of the ferromagnetism^32,33^. At the same time, both longitudinal and transverse resistivity are sensitive to unusually small in-plane magnetic fields, an effect that is not yet understood.

## Results

Devices were fabricated using established techniques^1,34^ (see Methods), and patterned into Hall bar geometries to measure *R*_*xx*_ and *R*_*xy*_. Figure 1a shows a schematic of the device architecture and measurement scheme. Multiple voltage probe pairs were measured in each device, with similar behaviour across most pairs (see SI). Mixing between *R*_*xx*_ and *R*_*xy*_ has been minimized in the data as presented by reporting the field symmetrized longitudinal resistance, henceforth labelled *ρ*_*xx*_, and the anti-symmetrized transverse resistance, *ρ*_*xy*_ (see Methods). Reported values of *B*_⊥_ have been adjusted to reflect flux trapping in the superconducting magnet (see SI). Similar behaviour is seen in two devices (D1 and D2), with twist angles *θ* ≈ 1.31° and *θ* ≈ 1.34° respectively; these were the only two devices with twist angle near 1.3° that were measured.

A typical resistivity map over top- and back-gate is shown in Fig. 1d, plotted with respect to *n* and *D*. Insulating stripes at *n* = 0 reflect the separation of conduction from valence band by finite *D*, while the insulating stripe around *n* = 4 × 10^12^ cm^−2^ reflects full filling of the first conduction band. Given the 4-fold degeneracy of the band (spin and valley), full filling is achieved at four electrons per moiré cell (*ν* = 4), allowing the twist angle to be calculated and the relation between *n* and *ν* determined (see Methods). Near *D* ~ ± 0.4 V/nm, numerical calculations indicate a moiré band that is nearly flat and isolated both from lower and upper bands. Figure 1b shows single-particle and self-consistent Hartree calculations of the moiré band structure for the parameters matching experimental conditions (see caption), illustrating the flatness of the band especially in the self-consistent calculation that highlights the role of interactions^19,35^.

Setting *D* near ± 0.4 V/nm in the device, a strong insulating state at *ν* = 2 is clearly visible, consistent with previous reports^21,22,24^, with surrounding 'halo' regions of higher resistivity sharply separated from a lower-resistance background. The relatively narrow range of ∣*D*∣ over which the halos appear is explained by the importance of an isolated narrow band for strong interaction effects: at too-small values of ∣*D*∣ there is almost no gap between conduction and valence band, while for too-large *D* the first moiré band overlaps with the second. The high quality of these devices is demonstrated by the the strong insulating state at *ν* = 3 even at **B** = 0; this signature is known to emerge only over a narrow range of twist angle *θ* ~ 1. 3° ^24^ where the correlations are maximum.

**Fig. 2:**
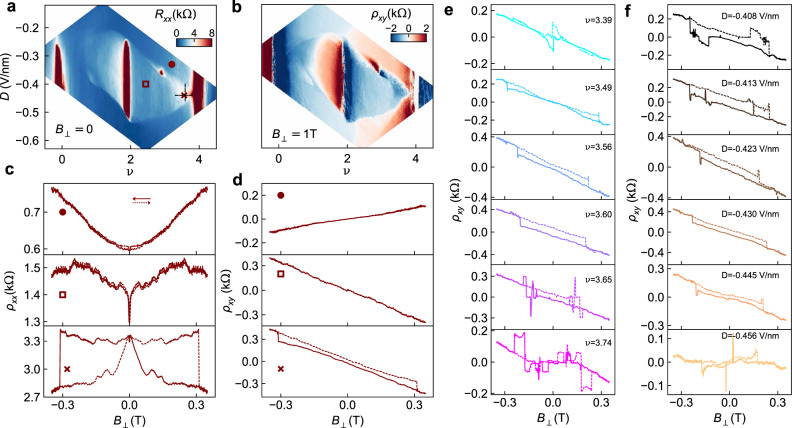
Out-of-plane magnetoresistance. **a** *R*_*xx*_ as function of *ν* and *D* showing the correlated insulating states at *ν* = 2 and *ν* = 3, appearing in red by the colour scale chosen here, surrounded by a halo region that is metallic but higher resistance (light blue) than the surrounding areas (dark blue). **b** Anti-symmetrized Hall resistance, *ρ*_*xy*_, for ∣*B*_⊥_∣ = 1T. The Hall resistance changes sign at integer fillings and also at the boundary of the halo region. **c**, **d** Magnetic field dependence of the symmetrized longitudinal resistance, *ρ*_*xx*_(*B*_⊥_), and anti-symmetrized Hall resistance, *ρ*_*xy*_(*B*_⊥_), for three values of {*ν*, *D*} marked in **a**. *B*_⊥_ is swept back and forth, shown with solid (positive to negative) and dashed (negative to positive) lines. **e**, **f** *ρ*_*xy*_(*B*_⊥_) as *B*_⊥_ is swept back and forth (solid/dashed) for (**e**) varying fillings *ν* = 3.4 → 3.75 at fixed *D* = −0.43 V/nm, and (**f**) varying *D* = −0.4 → = −0.46 V/nm at fixed *ν* = 3.6. D1 at *T* = 20 mK for all.

Figure 2 a offers a higher resolution map of *R*_*xx*_ across the halo region at negative *D*, with insulating states at *ν* = 2 and *ν* = 3 clearly visible. More insight into the broken-symmetry metallic states within the halo may be obtained from the Hall resistance, shown for *B*_⊥_ = ± 1*T* in Fig. 2b. *ρ*_*xy*_ changes sign across *ν* = 2 within the entire halo, and also at *ν* = 3 across a narrow region at the low-*D* edge of the halo. This behaviour implies that the 4-fold band degeneracy is broken throughout the halo region, and fully lifted for a narrow range of *D*.

The most telling data comes from the magnetoresistance of the correlated metallic states. Figure 2c, d show *ρ*_*xx*_ and *ρ*_*xy*_ as *B*_⊥_ is swept from 350 mT to −350 mT and back, comparing data for three {*ν*, *D*} pairs, indicated by markers in Fig. 2a. These datasets are chosen to illustrate three characteristic behaviours observed within the {*ν*, *D*} map. Outside the halo region (•), longitudinal magnetoresistance is weak and the data are independent of sweep direction, consistent with the behaviour of non-interacting metals. Within the halo but far from *ν* = 4 (□), *ρ*_*xx*_ shows an additional strong but narrow positive magnetoresistance, but *ρ*_*xy*_ is again nearly linear and neither *ρ*_*xx*_(*B*_⊥_) nor *ρ*_*xy*_(*B*_⊥_) depend on sweep direction. Within the halo and near *ν* = 4 ( × ), both *ρ*_*xy*_ and *ρ*_*xx*_ are strongly hysteretic, a clear indication of spontaneously broken time reversal symmetry giving rise to ferromagnetism with coercive fields near 0.3 T (bottom panels in Fig. 2c,d). AHE was also observed for the halo at positive *D*, though the hysteresis was less pronounced (see SI).

The range of *ν* over which ferromagnetic AHE signals appear is illustrated in Fig. 2e, showing data along the *D* = − 0.43 V/nm line in Fig. 2a. Close to the single-particle insulator (*ν* > 3.75), multiple jumps are seen in up-and-down magnetic field sweeps, indicating multi-domain switching behaviour; this behaviour continues deep into the insulating state. Throughout the range 3.5 ≲ *ν* ≲ 3.7, the hysteresis loop was wide and clean with nearly constant coercive field, then below *ν* ~ 3.4 both the width and height of the hysteresis loop collapsed. The reduced width of the loops indicates lower coercive fields for *ν* ≲ 3.4. The reduced height may result from reduced valley polarization or smaller Berry curvature: numerical calculations indicate that the strongest Berry curvature is near *k*_*x*_ = *k*_*y*_ = 0, corresponding to the highest energy (last-filled) states in the moiré band(Fig. 1c). For *ν* < 3.3, no *R*_xy_ hysteresis loops of the form shown in Fig. 2d, f were seen, though evidence of first-order domain flips was visible in the narrow region of gate voltage immediately adjacent to the *ν* = 3 insulating state (see SI). Taken together, the data are consistent with valley polarization originating from the *ν* = 3 state but only developing into AHE with strong Berry curvature above *ν* = 3.4.

Interestingly, AHE hysteresis loops were nearly independent of *D* within the halo, although outside the halo they were absent. The data in Fig. 2f represent the evolution of the AHE at fixed *ν* = 3.6, varying the displacement field from *D* = − 0.4 V/nm to *D* = − 0.46 V/nm (Fig. 2f). This uniformity contrasts with the very narrow range of *D* over which the *ν* = 3 insulating state is observed.

Having established a ferromagnetic AHE close to full filling of the moiré band, we turn to the question of whether spin or valley symmetry breaking is responsible for the observed effect. Previous reports of spin polarization in the *ν* = 2 insulator and presumably in the surrounding metallic states might suggest spin ferromagnetism as a possible source for the AHE, but the weak spin-orbit interaction^36^ of graphene makes this explanation less likely. Experimental input into this question is obtained from a comparison of in-plane and out-of-plane magnetoresistance: whereas the valley degree of freedom couples primarily to out-of-plane field, spins are expected to couple to total magnetic field with *g* = ~2.

**Fig. 3:**
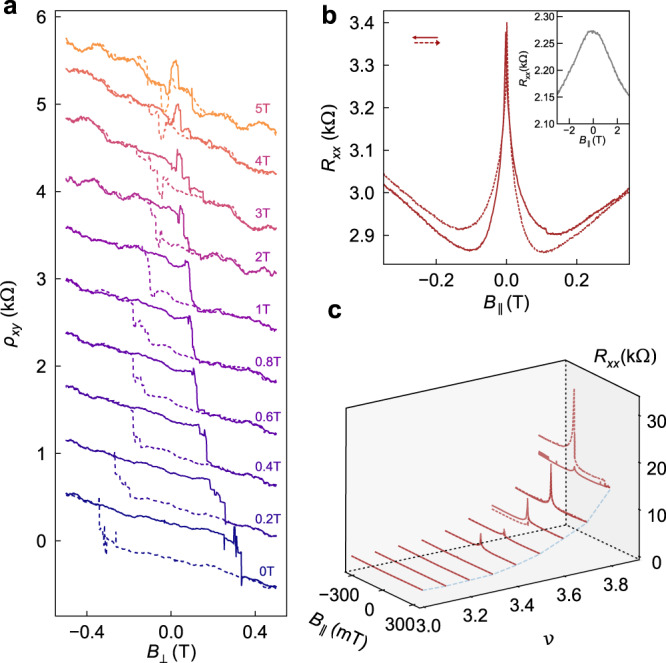
Anisotropic magnetoresistance. **a** Hysteresis in *ρ*_*xy*_(*B*_⊥_) for fixed in-plane magnetic fields (*B*_∥_) up to 5 T. The curves are offset by 600 Ω for clarity (D2, *ν* = 3.57, *D* = −0.43 V/nm, *T* = 100 mK). **b** A sharp peak in *R*_xx_(*B*_∥_) is observed at *B*_∥_ = 0 throughout the AHE region. The slight offset in *R*_xx_, comparing up and down traces away from *B* = 0, is caused by additional heating of the sample when sweeping the magnet away from zero field. Inset: finer scan just over the peak illustrates rounding on the *B*_∥_ = 1 mT field scale (D1, *ν* = 3.43, *D* = −0.42 V/nm, *T* = 100 mK). **c** Evolution of the *R*_xx_(*B*_∥_) magnetoresistance with *ν*. The peak appears immediately upon entering the AHE region (*ν* ≳ 3.4). Difference between up and down traces illustrates irreproducibility of *B*_∥_ sweeps at low field (D2, *D* = −0.43 V/nm, *T* = 300 mK, *B*_⊥_ = 0).

Figure 3 explores the effect of *B*_∥_ in more detail. A clear illustration of the anisotropic nature of the AHE comes from Fig. 3a, where *B*_⊥_ hysteresis loops (±450 mT) are shown for increasing fixed *B*_∥_. A particularly robust AHE in D2 exhibits a small hysteresis loop in *B*_⊥_, with coercive field around ~50 mT, even when *B*_∥_ is held at 5T. (Equivalent data for D1 show *B*_⊥_ hysteresis persisting above *B*_∥_ ~ 1 T, see SI.) The weak dependence of the AHE on the in-plane component of the magnetic field, up to *B*_∥_ of several Tesla, is reminiscent of the angle-dependent hysteretic AHE observed in twisted bilayer graphene aligned to *h*BN^33^. In that work, the AHE signature depended exclusively on the out-of-plane magnetic field component, leading to the conclusion that the AHE signal must be attributed to valley polarization. That conclusion was based on the understanding that valley order is only weakly sensitive to *B*_∥_.

## Discussion

In our measurements of tDBG, as in ref. 33, there is a qualitative distinction between phenomena driven by *B*_⊥_ and *B*_∥_. But the influence of *B*_∥_ is nevertheless very strong—much stronger (especially for small *B*_∥_) than in ref. 33. Figure 3b, c provide illustrations of this extreme *B*_∥_ magnetoresistance, which was observed in both devices. Throughout the AHE region an anomalously sharp negative *B*_∥_-magnetoresistance was observed (Fig. 3b), with a rounding around zero field on the *B*_∥_ ~ 1 mT scale that is orders of magnitude smaller than what might be expected when comparing *gμ*_*B*_*B*_∥_ to *k*_*B*_*T* (*k*_*B*_*T*/*gμ*_*B*_ ~ 100 mT at 100 mK for *g* = 2). Examining a sequence of *B*_∥_ sweeps for increasing *ν*, along a line of fixed *D* = − 0.43 in the centre of the halo (Fig. 3c, c.f. Fig. 2e), the peak appears immediately upon entering the AHE region (*ν* ≳ 3.4) and grows with larger values of *ν*. Beyond the consistently-observed peak in *R*_xx_(*B*_∥_), however, the details of *B*_∥_ magnetoresistance in the AHE region were less repeatable from scan to scan than the hysteresis in *B*_⊥_ that is the primary subject of this work. The *B*_∥_ scans in Fig. 3c are neither hysteretic nor exactly reproducible, in marked contrast to the AHE hysteresis loops reported elsewhere in this work, which retrace scan after scan.

The striking effects of small *B*_∥_ seen in Fig. 3 have not, to our knowledge, been reported before; they appear in both devices, and only inside the AHE regions. These effects may be due to the coupling, albeit weak, of *B*_∥_ to valley order^10^. Even though this coupling is small, proportional to the thickness of the double bilayer, it competes with a valley anisotropy that is poorly understood at present and likely also to be weak. Alternately, *B*_∥_ could influence the AHE via spin-orbit couping as reported recently in twisted (single) bilayer graphene with spin-orbit interaction enhanced by proximity to WSe_2_^37^. Although spin-orbit coupling was not intentionally enhanced in our devices, this possibility cannot be excluded. A more complete study of the *B*_∥_-induced effects in tDBG will be the subject of future work.

**Fig. 4:**
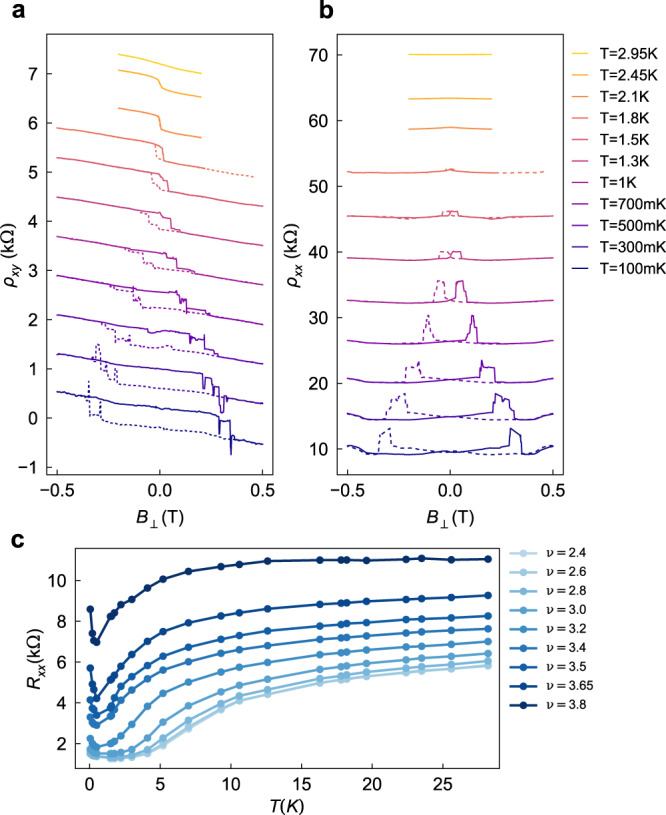
Temperature dependence of AHE. **a** Temperature dependence of hysteresis loop *ρ*_*xy*_(*B*_⊥_) (D2, *ν* = 3.57, *D* = −0.43 V/nm). Curves are offset by 800 Ω for clarity. **b** Temperature dependence of *ρ*_*xx*_(*B*_⊥_); curves offset by 6 *k*Ω for clarity. **c** Temperature dependence *R*_*xx*_(*T*) at **B** = 0 in the halo region for *ν* in the range *ν* = 2.4–3.8 (D1, *D* = −0.43 V/nm).

The AHE signatures seen in Fig. 2 persist up to 1.8 K in D2 (similar in D1), illustrating the relatively large energy scales associated with valley symmetry breaking in this system. Figure 4a, b show the temperature-induced collapse of the hysteresis loop via shrinking coercive field for *ρ*_xy_ _xx_ and *ρ*_xx_ _xy_ respectively. Early measurements of tDBG noted a step-like rise in *R*_xx_ with temperature within the halo region, which has been attributed to temperature-induced collapse of broken symmetry states^24^. An analogous rise in *R*_xx_ around 7  K is observed in our measurements away from the AHE region (*ν* < 3.2, Fig. 4c). The onset of AHE with increasing *ν* is correlated with a sharp decrease in the transition temperature, consistent with the Kelvin-scale collapse of the symmetry-broken state that gives rise to the hysteresis in Fig. 4a,b. The gradual shift of the transition temperature with increasing *ν* in Fig. 4, instead of the appearance of a second symmetry breaking transition in the AHE region, supports the notion of a correlated low-*T* ground state with coupled spin and valley order^38,39^.

In conclusion, we have observed an AHE, signifying orbital magnetic order, in AB-AB stacked tDBG. The ferromagnetic state occurs only within the strongly interacting ‘halo’ regions of the (*ν*, *D*) plane. Strong magnetic anisotropy suggests valley ferromagnetism, while in-plane field signatures indicate a complex interplay of broken spin/valley symmetries in the correlated metallic state of tDBG.

## Methods

### Device fabrication

High quality AB-AB stacked tDBG devices were fabricated using the 'modified' tear and stack technique^1,40^. First, a large bilayer graphene (BLG) flake ~ 70 μm was exfoliated on a Si/SiO_2_ substrate with 285nm oxide. The BLG flake was mechanically precut into two pieces using a sharp ~ 1 μm diameter tungsten dissecting needle which was attached to the micromanipulator in our transfer setup. Using a stamp made of polybisphenol carbonate (PC) on polydimethylsiloxane (PDMS), the top *h*BN layer was picked up at *T* = 100 °C. Next, the BLG flake was picked up at *T* = 30 °C. The stage was rotated to 1.36°, followed by picking up the second BLG at *T* = 30 °C. Then the bottom *h*BN layer was picked up at *T* = 100 °C, followed by graphite at *T* = 110 °C. The sequence of stacking from top to bottom is *h*BN, tDBG, *h*BN and graphite. Finally, the entire stack was deposited on a Si/SiO_2_ substrate at *T* = 175 °C. Top gate was defined using electron beam lithography (EBL), followed by deposition of Cr/Au (5 nm/50 nm). Edge contacts were formed by etching the stack in CHF_3_:O_2_ plasma, followed by deposition of Cr/Au (5 nm/80 nm) at a base pressure of ~ 1 × 10^−7^mbar. Finally, another step of EBL and etching was done to pattern the device in a Hall bar geometry. The top and bottom *h*BN thickness was determined by atomic force microscope.

### Transport measurements

The transport measurements were performed in four terminal configuration, using standard lock in (SRS830) techniques at *f* ~ 13.3 Hz with a current bias *I* = 1 nA. The experiment was carried out in a Bluefors cryogen-free dilution refrigerators equipped with a 3-axis magnet enabling simultaneous control of in- and out-of-plane magnetic fields. Except where noted, measurements were carried out at the nominal base temperature of the refrigerator, with the mixing chamber at 10–15 mK, but heating due to magnetic field sweeps raised the sample temperature up to 50–200 mK depending on sweep conditions.

Twist angle, *θ*, was calculated from the density corresponding to full filling of the moiré miniband *n*(*ν* = ± 4) = 8${\theta }^{2}/\sqrt{3}{a}^{2}$, where *a* = 0.246 nm is the lattice constant of graphene. In D1, *ν* = ± 4 corresponds *n* = ± 4.05 × 10^12^cm^−12^, indicating a twist angle *θ* ≈ 1.31°. In D2, *ν* = ± 4 corresponds *n* = ± 4.16 × 10^12^cm^−12^ giving *θ* ≈ 1.34°.

Top gate (*V*_*tg*_) and bottom gate (*V*_*bg*_) voltages were used to control the charge carrier density (*n*) and the displacement field (*D*) independently. The density is given by *n* = (*C*_*bg*_*V*_*bg*_ + *C*_*tg*_*V*_*tg*_)/*e* and the displacement field is given by *D* = ∣*C*_*bg*_*V*_*bg*_ − *C*_*tg*_*V*_*tg*_∣/2*ϵ*_0_, where *C*_*bg*_ (*C*_*tg*_) are the bottom(top) gate capacitances per unit area, *e* is the electronic charge and *ϵ*_0_ is the vacuum permittivity. We label the field symmetrized (anti-symmetrized) data for *R*_*xx*_ (*R*_*xy*_) as *ρ*_*xx*_ (*ρ*_*xy*_), where ${\rho }_{xx}(B,\uparrow )=\frac{{R}_{xx}(B,\uparrow )+{R}_{xx}(-B,\downarrow )}{2}\frac{W}{L}$, ${\rho }_{xx}(B,\downarrow )=\frac{{R}_{xx}(B,\downarrow )+{R}_{xx}(-B,\uparrow )}{2}\frac{W}{L}$ and the anti-symmetrized Hall resistance ${\rho }_{xy}(B,\uparrow )=\frac{{R}_{xy}(B,\uparrow )-{R}_{xy}(-B,\downarrow )}{2}$, ${\rho }_{xy}(B,\downarrow )=\frac{{R}_{xy}(B,\downarrow )-{R}_{xy}(-B,\uparrow )}{2}$, using *↑*, *↓* to indicate the magnetic field sweep direction.

## Supplementary information

Supplementary Information

## Data Availability

Additional information related to this work is available from the corresponding author upon reasonable request. Source data are provided with this paper.

## Source data

Source Data

## Code availability

The code used for the purposes of analyzing data is also available from the corresponding author upon request.
