# Supplementary material for: Spontaneous time-reversal symmetry breaking in twisted double bilayer graphene
Source: Nat Commun. 2022 Oct 29;13:6468. doi: 10.1038/s41467-022-34192-x (PMC9617879; doi:10.1038/s41467-022-34192-x)
Supplement: Supplementary file 1 — Supplementary Information [file 41467_2022_34192_MOESM1_ESM.pdf]

Supplementary Material for "Spontaneous time-reversal symmetry breaking in twisted double bilayer graphene"

Manabendra Kuiri[1],* Christopher Coleman[1], Zhenxiang Gao[1], Aswin Vishnuradhan[1], Kenji Watanabe[2], Takashi Taniguchi[3], Jihang Zhu[4], Allan Macdonald[4], and Joshua Folk[1]†

[1] *Department of Physics and Astronomy & Stewart Blusson Quantum Matter Institute, University of British Columbia, Vancouver BC, Canada V6T 1Z4*

[2] *Research Center for Functional Materials, National Institute for Materials Science, Namiki 1-1, Tsukuba, Ibaraki 305-0044, Japan*

[3] *International Center for Materials Nanoarchitectonics, National Institute for Materials Science, Namiki 1-1, Tsukuba, Ibaraki 305-0044, Japan and*

[4] *Physics Department, University of Texas at Austin, Austin TX USA 78712*

* koolmanab@gmail.com

† jfolk@physics.ubc.ca

I. Device Fabrication

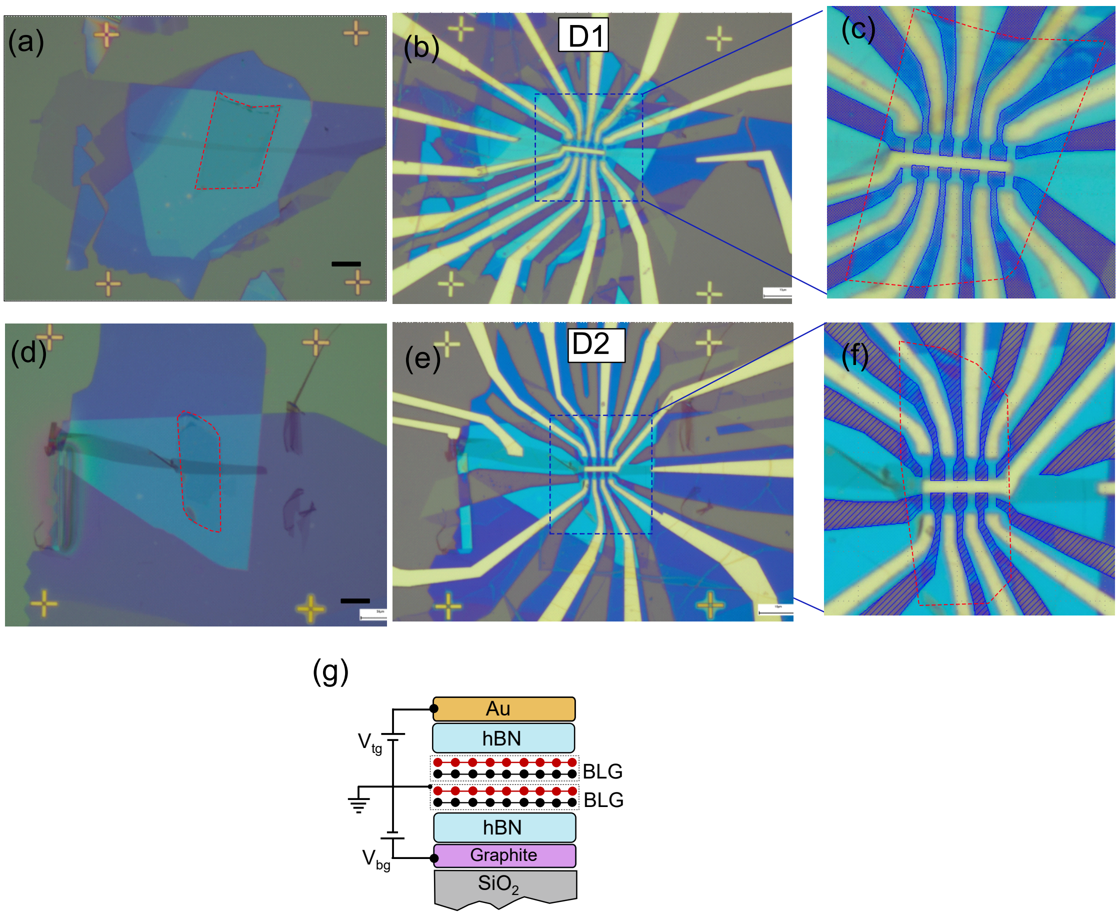

FIG. S1. **Device Fabrication.** (a) Optical image of the device D1 consisting of hBN/tDBG/hBN/graphite with a twist angle $\theta \approx 1.31^\circ$ after stacking. tDBG boundary highlighted by red dashed line Scale bar is 7μm. (b) Optical image of the device D1, after nanofabrication. (c) Zoomed-in optical image of the device D1, showing the contacts. The etched region has been shown in blue. (d) Optical image of second device D2 consisting of hBN/tDBG/hBN/graphite with $\theta \approx 1.34^\circ$. Scale bar is 7$\mu$m. (e) Optical image of the device D2, after nanofabrication. (f) Zoomed-in optical image of the device D2, showing the contacts. The etched region has been shown in blue. In both devices, the graphite layer serves as bottom gate, V_{bg}. The samples were not intentionally aligned to hBN, and accidental alignment can be ruled out because both devices (D1 and D2) show a similar behavior, and transport characteristics were the same for both positive and negative D.[1]. (g) Cross section schematic of the devices.

II. Sample uniformity and device characterization.

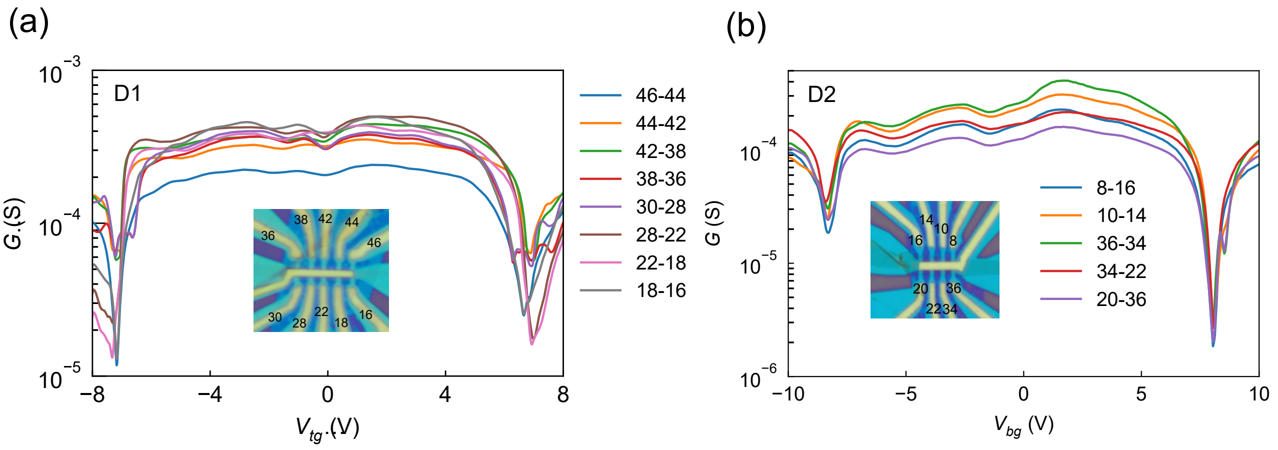

FIG. S2. **Sample uniformity and device characterization.** (a) Two wire conductance measurements between all neighbouring contact pairs at T =4K for device D1. The inset shows the optical image of the device with contact pairs labelled. Here, an ac excitation voltage of $V_{ac} = 100\mu$V was applied and the current was measured via the other contact, while all other contacts remained floating. The difference between the position of the moiré peak is used to estimate the local twist angle. The variation of $\delta V_{tg}(\delta n)$ for different contact pairs for D1 is $\delta n \sim 9 \times 10^{-10}$cm^{-2}, which translates to an angle inhomogeneity of $\delta\theta \sim 0.02°$ over a length of 12μm. The slightly anomalous pair 46-44 was not used in this experiment. (b) Two wire conductance measurement for device D2 at T =4K. The twist angle inhomogeneity in this device, D2 is $\delta\theta \sim 0.01°$. The corresponding contact pairs are labelled in the inset.

III. Four terminal measurements (D1).

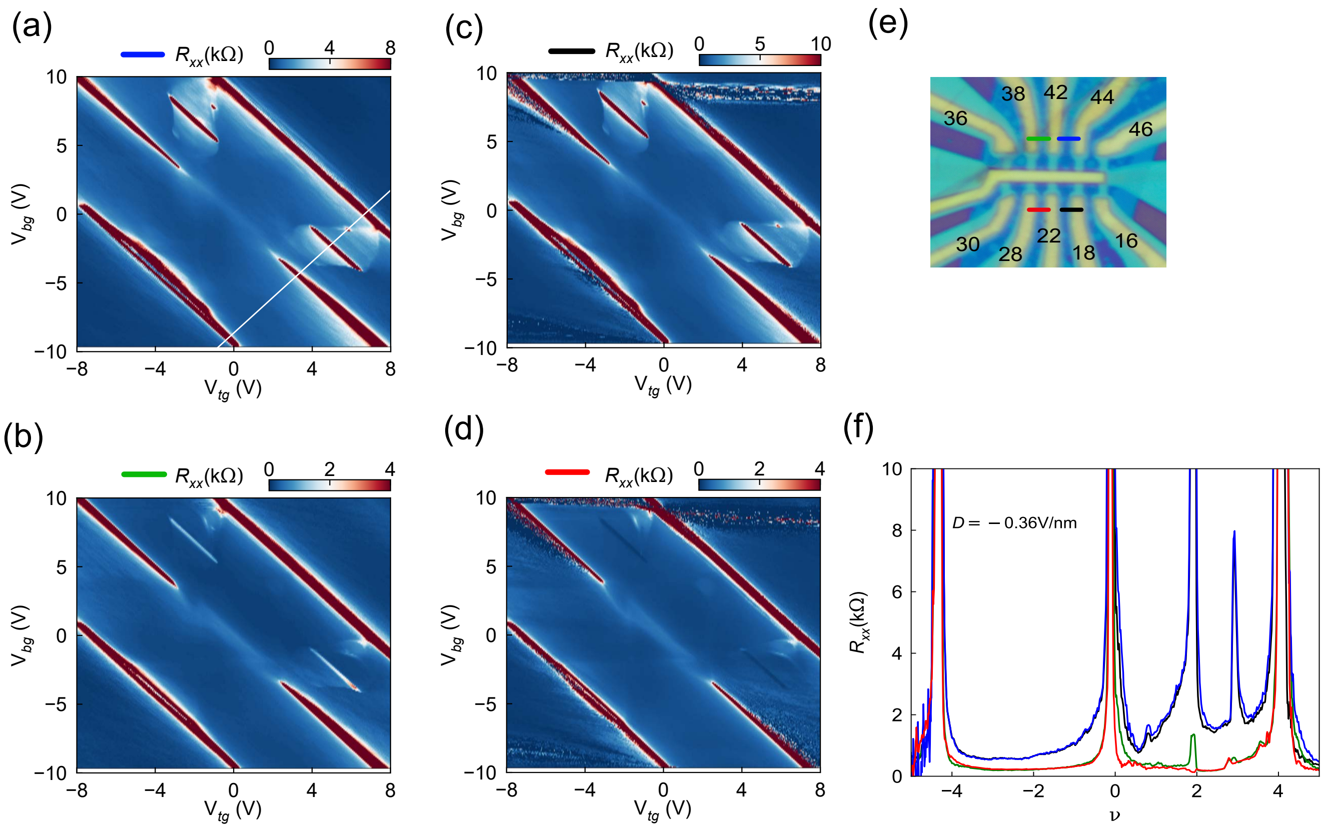

FIG. S3. **Comparison for different voltage probes: device 1 at $B = 0$.** (a)-(d) 2D colorplot of the measured Resistance as a function of topgate voltage (V_{tg}) and backgate voltage (V_{bg}) at $B = 0$, and T=20mK, for device D1 for several pair of contacts color coded in (e). The data in presented in Fig.1d of the manuscript, transformed to axes of ν and D, is from this figure panel (a). (f) Four terminal resistance as a function of filling factor, ν at $D = -0.36$ V/nm for several voltage probes, color coded in the inset. Here, current was injected in contact 16, and contact 30 was grounded. Most of the contact pairs show insulating states at filling $\nu = 0, 2, 3, \pm 4$, while the contact pair marked in red showed a suppression of the correlated insulating state at $\nu = 2$, with a resistance $\sim 220\Omega$. The white dashed line in (a) represents the line trace at $D = -0.36$ V/nm in the 2D map.

IV. Four terminal measurements (D2).

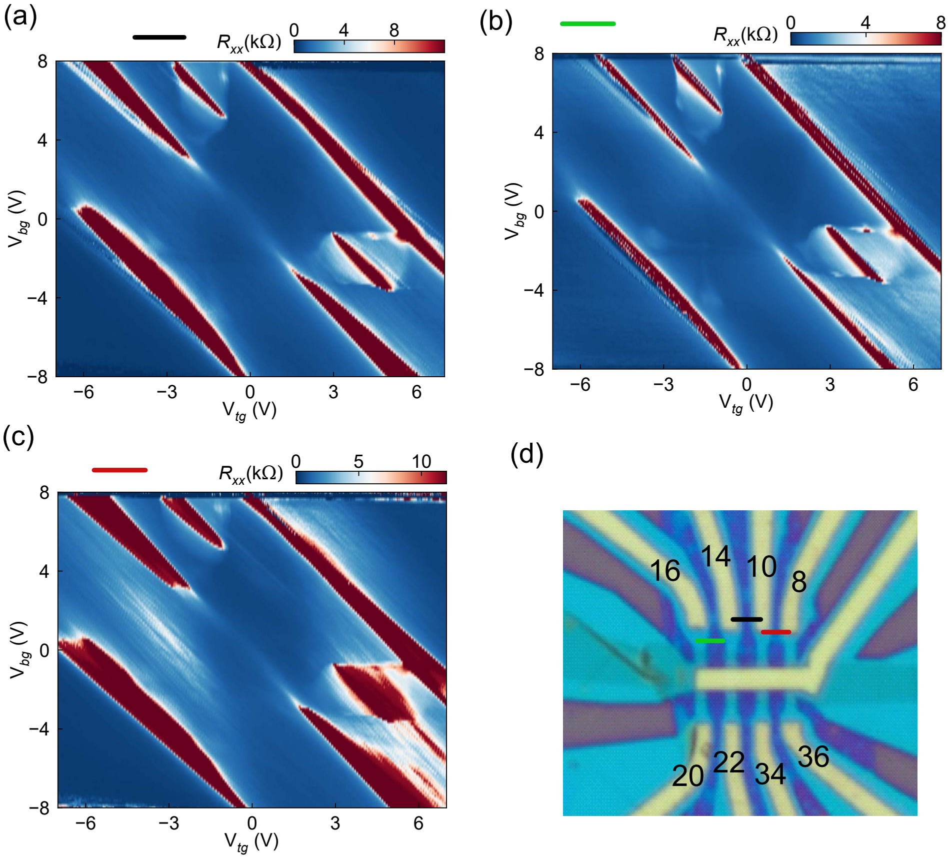

FIG. S4. **Comparison for different voltage probes: device D2 at** $B = 0$**.** (a)- (c) 2D colorplot of the measured resistance as a function of topgate voltage (V_{tg}) and backgate voltage (V_{tg}) at $B = 0$, and T=20mK, for several voltage pairs for device D2. The corresponding voltage pairs are color coded in (d).

V. Hysteresis in different voltage probes: device D1

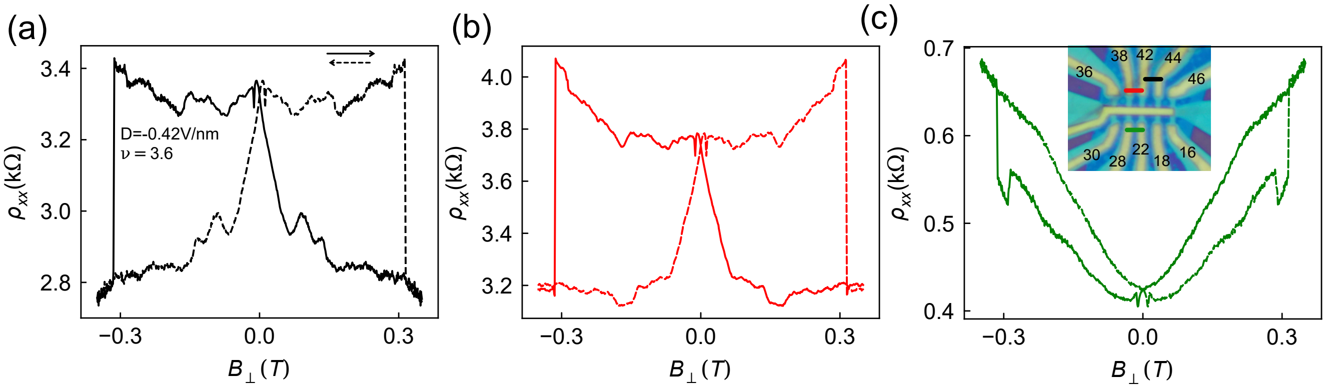

FIG. S5. **Comparison for different voltage probes: device D1.** (a) ρ_{xx} as a function of out of plane magnetic field for $\nu = 3.6$, $D = -0.42$V/nm, where the magnetic field is swept back and forth for three voltage probes color coded in the inset of (c).

VI. Hysteresis in different voltage probes: device D2

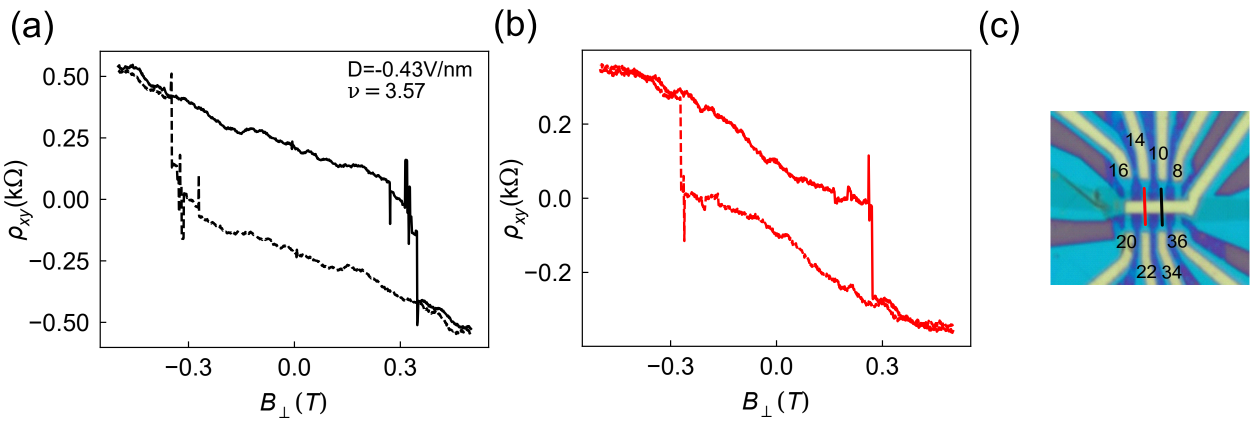

FIG. S6. **Comparison for different voltage probes: device D2.** (a) and (b) ρ_{xy} as a function of out of plane magnetic field for $\nu = 3.57$, $D = -0.43\text{V/nm}$, where the magnetic field is swept back and forth, for the contact pairs color coded in (c).

VII. Symmetrization and Anti-symmetrization of the measured longitudinal and Hall resistance, device D1

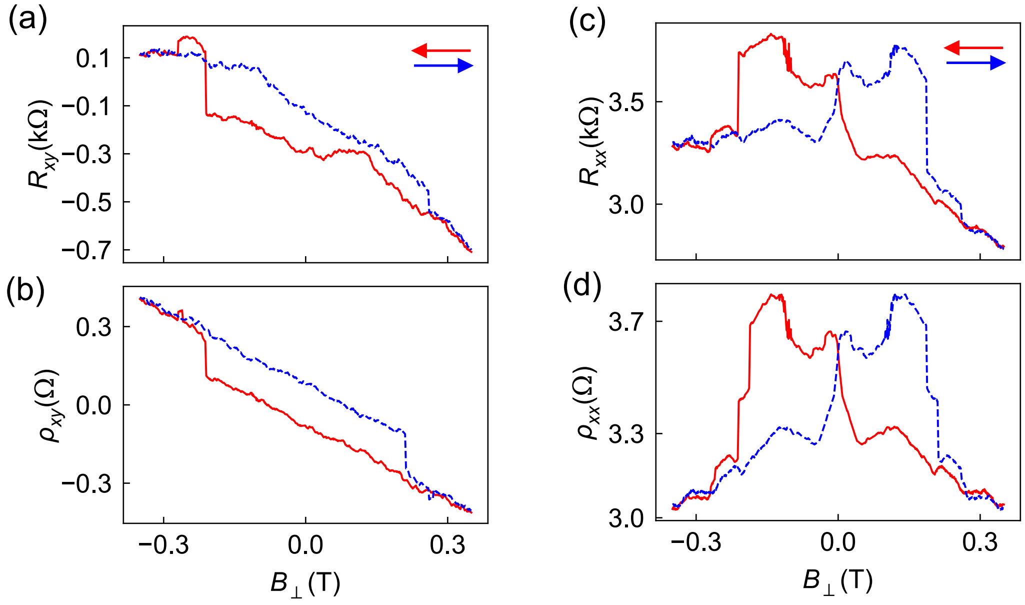

FIG. S7. **Symmetrization of R_{xx} and Anti-symmetrization of R_{xy}.** (a) Measured Hall resistance R_{xy} as a function of $B_\perp$, where the magnetic field is swept from $+B_\perp$ to $-B_\perp$ (red solid curve) and $-B_\perp$ to $+B_\perp$ (blue dashed curve). (b) Shows the corresponding anti-symmetrized Hall Resistance, ρ_{xy}. (c) Shows the measured longitudinal resistance R_{xx}. (d) Shows the symmetrized longitudinal resistance, ρ_{xx}. These data were taken at filling $\nu = 3.64$ and $D = -0.43\text{V/nm}$.

VIII. Symmetrization and Anti-symmetrization of the measured longitudinal and Hall resistance, device D2

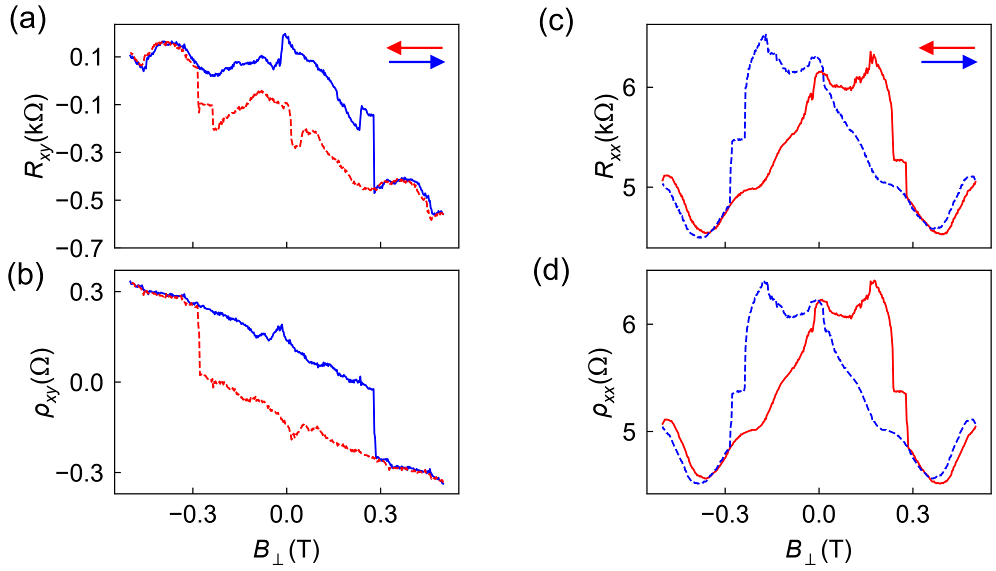

FIG. S8. **Symmetrization of R_{xx} and Anti-symmetrization of R_{xy}.** (a) Measured Hall resistance R_{xy} as a function of $B_\perp$, where the magnetic field is swept from $+B_\perp$ to $-B_\perp$ (red solid curve) and $-B_\perp$ to $+B_\perp$ (blue dashed curve). The corresponding anti-symmetrized Hall Resistance ρ_{xy} is shown in (b). (c) Shows the measured longitudinal resistance R_{xx} and the symmetrized longitudinal resistance, ρ_{xx} in (d). These data were taken at $\nu = 3.67$ and $D = -0.43$B/nm.

IX. Anomalous Hall effect for positive displacement field, $+D$, device D1

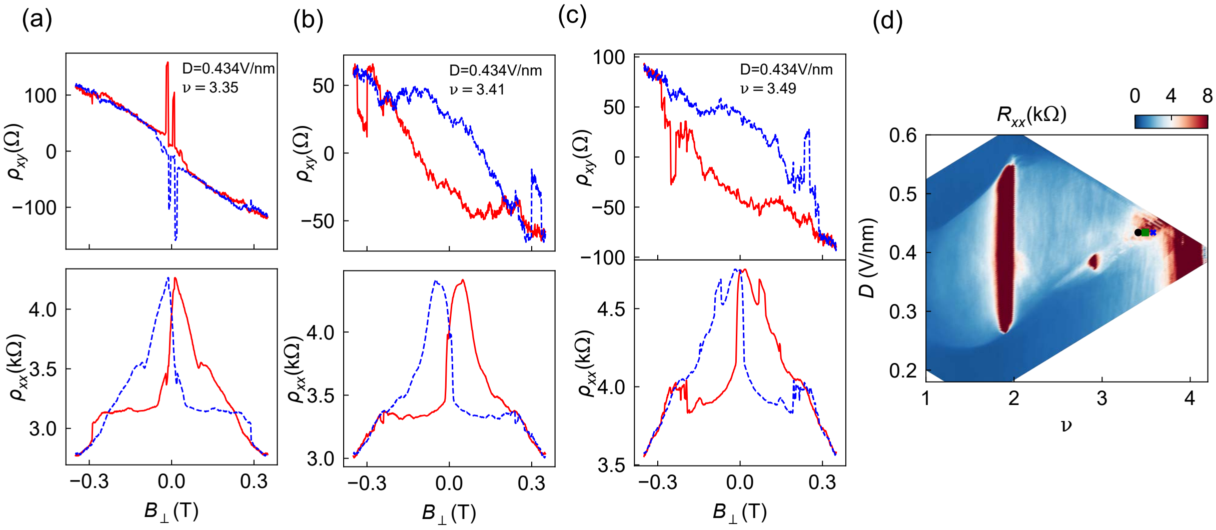

FIG. S9. **Anomalous Hall effect for positive displacement field ($+D$).** Due to pinching off of voltage probe contacts it was difficult to access the AHE part of the $+D$ halo region. As shown in Fig. S9d, the AHE corner is just at the edge of the measurable region (contacts pinched off outside this). Even in the limited range that was available, AHE was clearly observed. (a) - (c) Anti-symmetrized Hall resistance and symmetrized longitudinal resistances for for several values of ν and $+D$, with corresponding locations shown in (d).

X. Additional data: magnetic anisotropy for D1 and D2

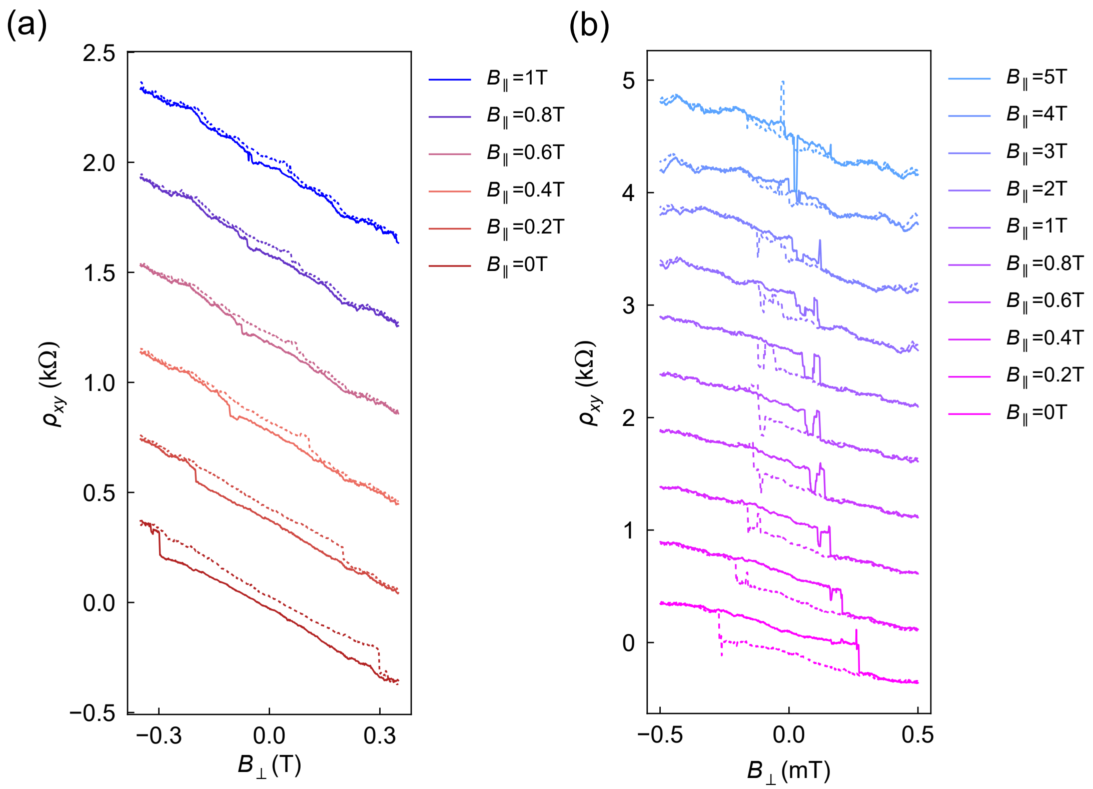

FIG. S10. **Magnetic anisotropy compared for samples D1 and D2.** (a) Out of plane hysteresis for several values of $B_\parallel$ for filling $\nu = 3.47$ and $D = -0.43$V/nm in device D1. (b) Out of plane hysteresis for several values of $B_\parallel$ at filling $\nu = 3.57$ and $D = -0.43$V/nm in device D2. This data is acquired for different pair of contacts as shown in Fig.3a of the main manuscript.

XI. Hall Resistance across the crescent extending to filling $\nu \approx 3$

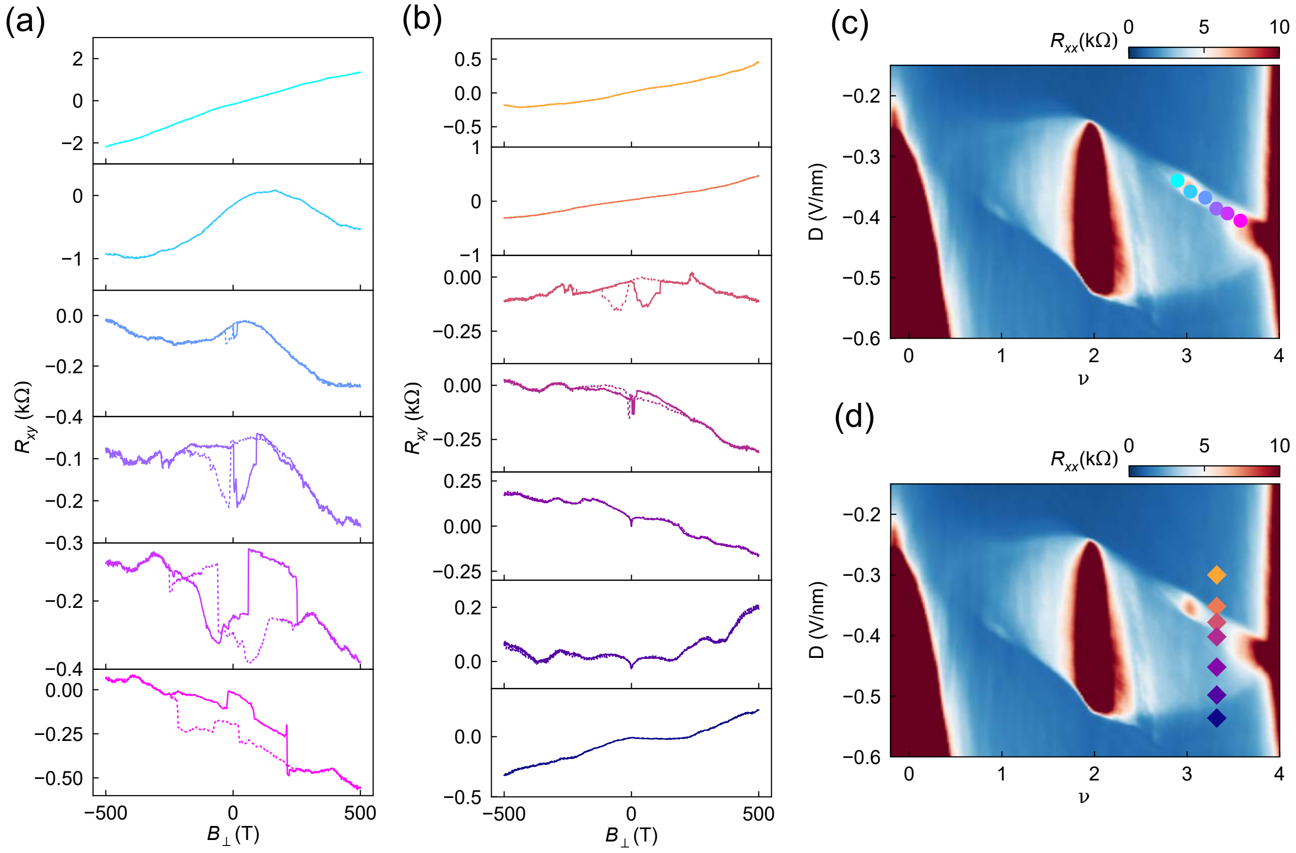

FIG. S11. **Hall resistance for sample D2 across the crescent.** (a) Measured hysteresis in Hall Resistance, R_{xy} for several values of filling ν and D across the edge of the Hall from filling $\nu \sim 3$ to the band edge. The corresponding points are marked in (c). (b) Measured hysteresis in Hall Resistance, R_{xy} for several values of D at fixed filling $\nu = 3.32$. The corresponding locations are color coded in (d).

XII. Absence of AHE close to filling $\nu \approx 0$

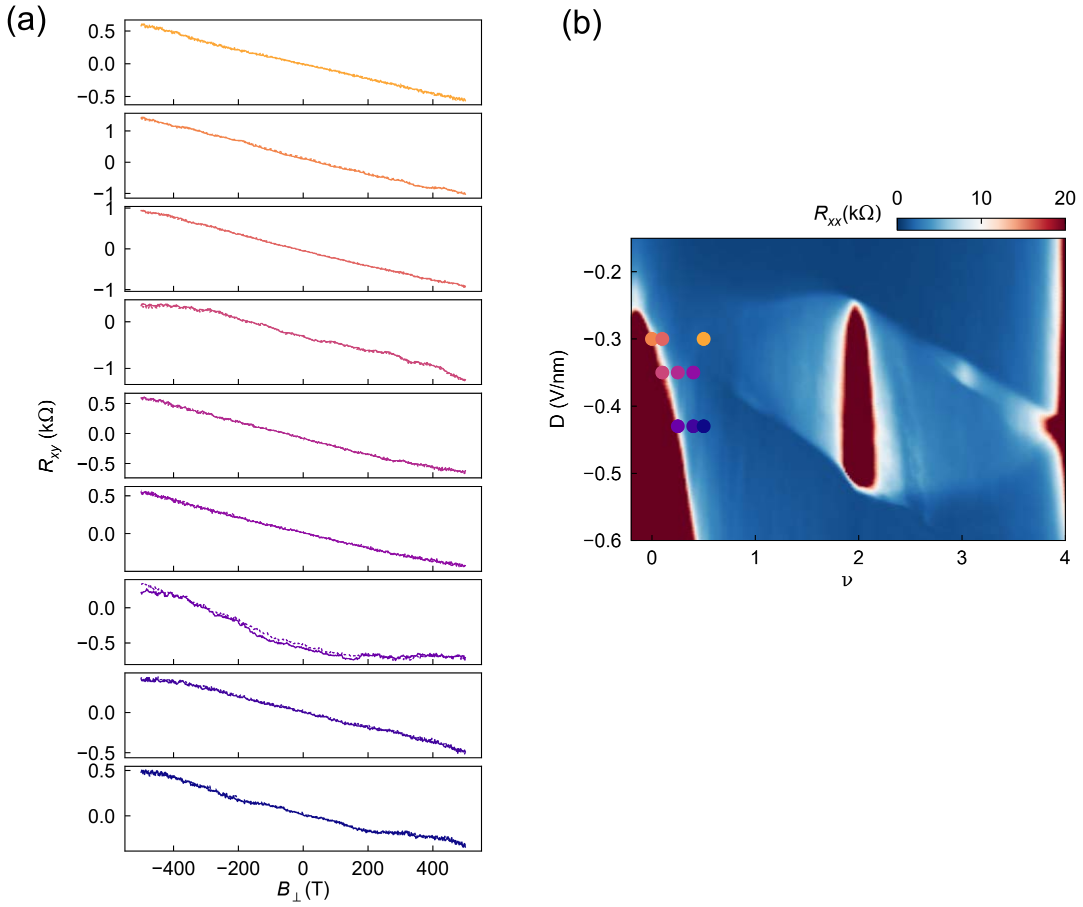

FIG. S12. **Hall resistance for sample D2 close to filling** 0. (a) Measured hysteresis in Hall Resistance, R_{xy} for several values of filling ν and D near filling $\nu \approx 0$. The corresponding locations are marked as colored circles in (b).

XIII. Evolution of sharp $R_{xx}(B_\parallel)$ peak with displacement field

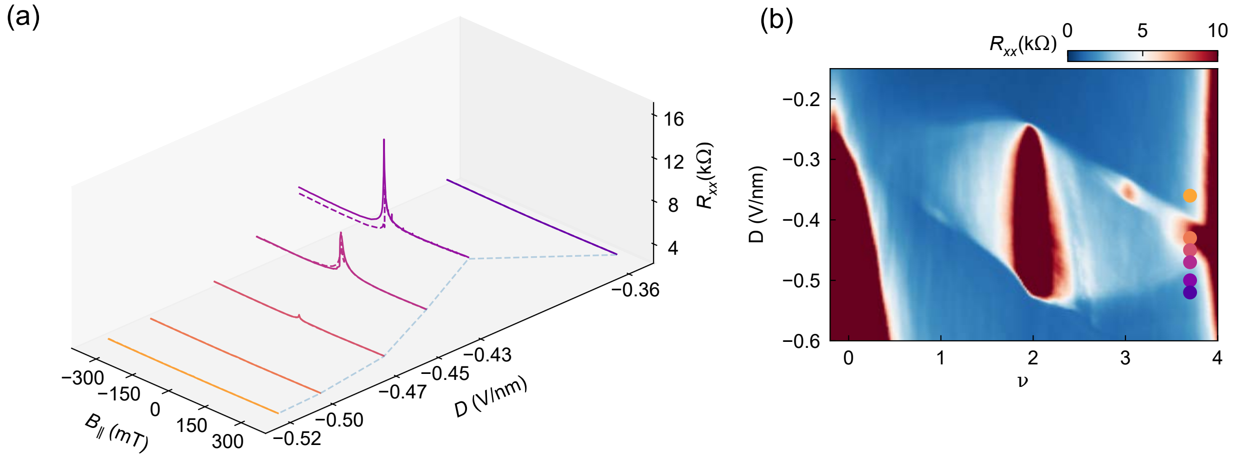

FIG. S13. (a) Measured R_{xx} as a function of inplane field, $B_\parallel$ swept back and forth at filling $\nu = 3.7$ for multiple values of D. corresponding locations are marked as colored circles in (b). Sample D2

XIV. Temperature dependence of the correlated insulating states, device D1

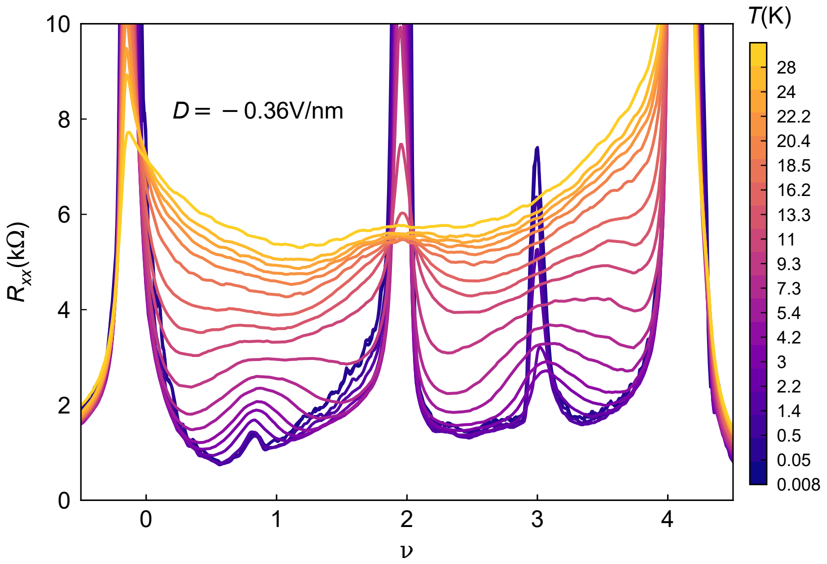

FIG. S14. **Temperature dependence.** Temperature dependence of the correlated insulating states, device D1. The correlated insulating state at $\nu = 2$ appears for T <15K, while the correlated insulating states for $\nu = 3$ appears for T <2K.

XV. Collapse of AHE with temperature, device D1

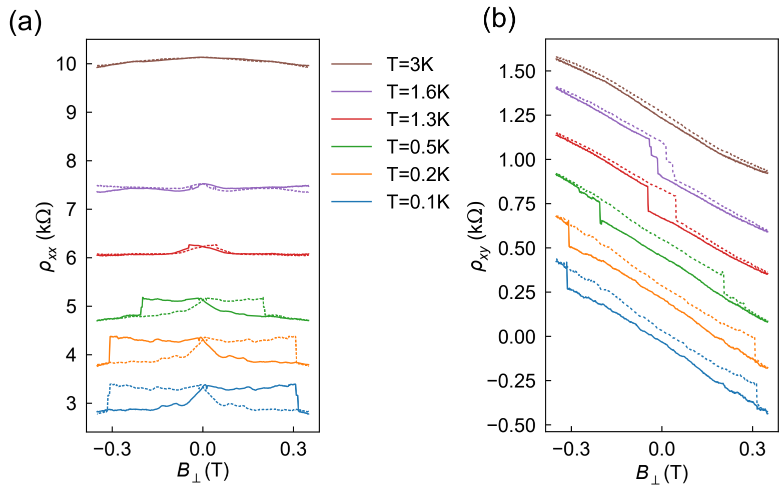

FIG. S15. **Temperature dependence of AHE, D1.** Temperature dependence of the hysteresis loop in ρ_{xy} for ν =3.58, and D=0.42 V/nm. Here each curves are shifted by 250Ω for clarity.(b) Shows the temperature dependence of ρ_{xx}, where each curves are offset by 1kΩ for clarity.

XVI. Fig 2, 3, 4 data without B correction

The magnetic field axis in Figs 2 and 3 is rescaled to account for trapped flux in the wires of our three-axis superconducting magnet.

The rescaling function maps B_{real}, the actual magnetic field estimated at the sample and the one plotted on the horizontal axes in the main text, to B_{mag}, the field set by the current in the magnet. The mapping depends on the sweep range and sweep direction, as the amount of trapped flux is greater for larger sweeps. The rescaling functions were estimated by examining R_{xy} obtained for up- and down- magnetic field sweeps outside of the halo regions. Because there was no hysteresis expected in resistivity data for these gate voltage settings, the rescaling function was defined by the offset needed to make up- and down-sweeps match up.

Scaling functions are given below, expressing B in mT:

- For ±500 mT scans: $B_{real} = B_{mag} \pm (12.5 - 12.5 * (B_{mag}/500)^2)$
- For ±350 mT scans: $B_{real} = B_{mag} \pm (11 - 11 * (B_{mag}/350)^2)$
- For ±200 mT scans: $B_{real} = B_{mag} \pm (8.5 - 8.5 * (B_{mag}/200)^2)$
- In all cases, the $+$ in the $\pm$ corresponds to downsweeps and the $-$ corresponds to upsweeps.
- We estimate a remaining inaccuracy less than ±2 mT in the actual value of B_{real} compared to B_{mag}.

For clarity, the raw (unshifted) data for Figs 2, 3 and 4 are shown below.

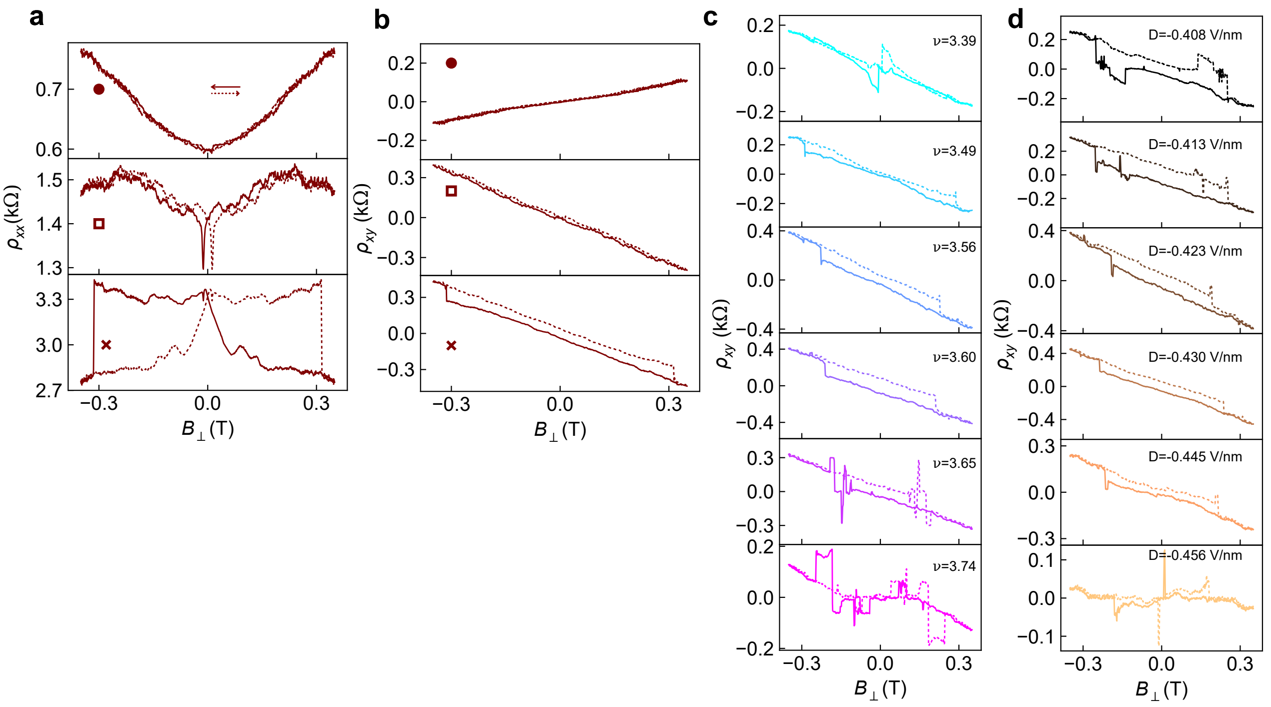

FIG. S16. **Fig 2 without magnetic field correction.** (a)-(d) Data of Fig 2 plotted against B_{mag}, that is, without magnetic field correction.

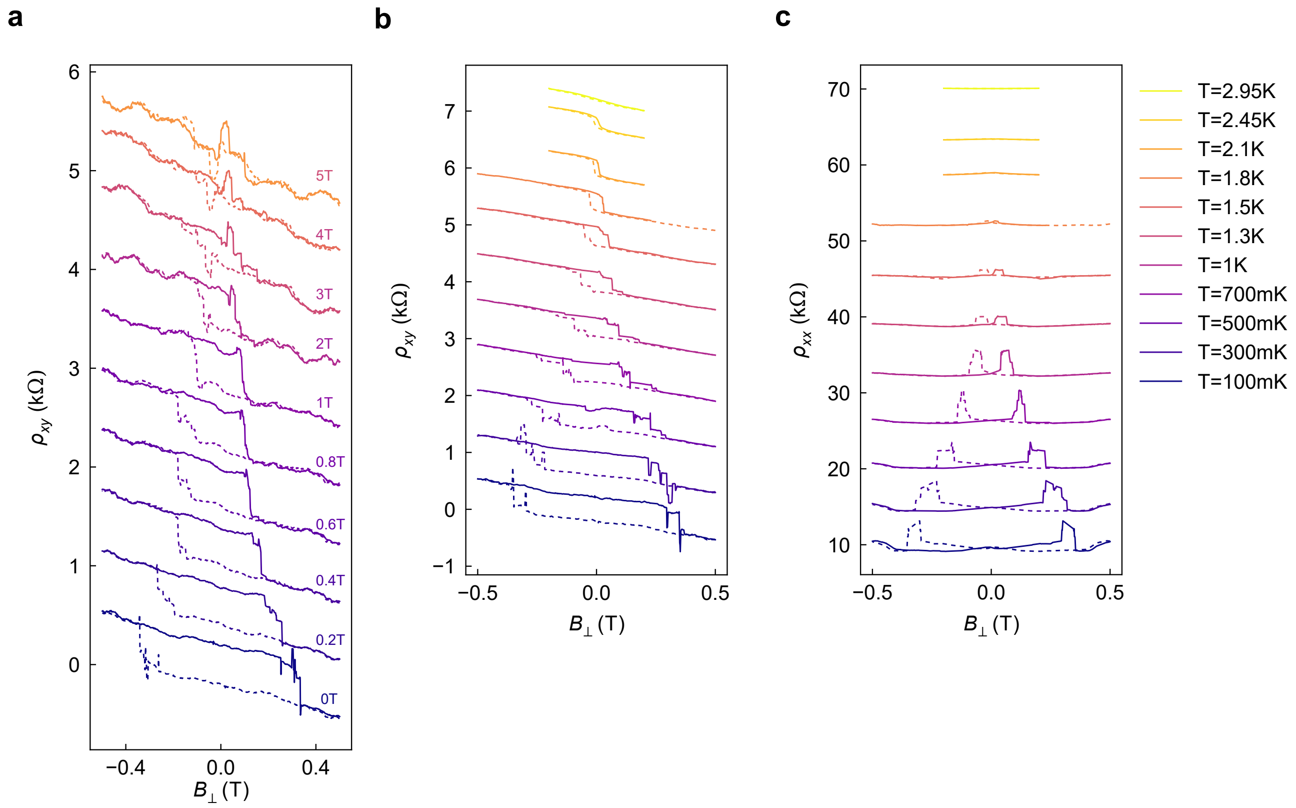

FIG. S17. **Fig 3 and 4 without magnetic field correction.** (a) Data of Fig. 3a plotted against B_{mag}, that is, without magnetic field correction. (b)-(c) Fig 4a and 4b of the main manuscript without magnetic field correction.

SUPPLEMENTARY REFERENCES

[1] M. He, J. Cai, Y.-H. Zhang, Y. Liu, Y. Li, T. Taniguchi, K. Watanabe, D. H. Cobden, M. Yankowitz, and X. Xu, Chirality-dependent topological states in twisted double bilayer graphene, arXiv preprint arXiv:2109.08255 (2021).
